# Unsupervised discrimination of patterns in spiking neural networks with excitatory and inhibitory synaptic plasticity

**DOI:** 10.3389/fncom.2014.00159

**Published:** 2014-12-15

**Authors:** Narayan Srinivasa, Youngkwan Cho

**Affiliations:** Center for Neural and Emergent Systems, Information and Systems Sciences Department, HRL Laboratories LLCMalibu, CA, USA

**Keywords:** spiking, STDP, learning, pattern discrimination, stability-plasticity dilemma, reservoir computing, balanced networks, basis sets

## Abstract

A spiking neural network model is described for learning to discriminate among spatial patterns in an unsupervised manner. The network anatomy consists of source neurons that are activated by external inputs, a reservoir that resembles a generic cortical layer with an excitatory-inhibitory (EI) network and a sink layer of neurons for readout. Synaptic plasticity in the form of STDP is imposed on all the excitatory and inhibitory synapses at all times. While long-term excitatory STDP enables sparse and efficient learning of the salient features in inputs, inhibitory STDP enables this learning to be stable by establishing a balance between excitatory and inhibitory currents at each neuron in the network. The synaptic weights between source and reservoir neurons form a basis set for the input patterns. The neural trajectories generated in the reservoir due to input stimulation and lateral connections between reservoir neurons can be readout by the sink layer neurons. This activity is used for adaptation of synapses between reservoir and sink layer neurons. A new measure called the discriminability index (*DI*) is introduced to compute if the network can discriminate between old patterns already presented in an initial training session. The *DI* is also used to compute if the network adapts to new patterns without losing its ability to discriminate among old patterns. The final outcome is that the network is able to correctly discriminate between all patterns—both old and new. This result holds as long as inhibitory synapses employ STDP to continuously enable current balance in the network. The results suggest a possible direction for future investigation into how spiking neural networks could address the stability-plasticity question despite having continuous synaptic plasticity.

## Introduction

A hallmark of biological systems is their ability to learn new knowledge while also exhibiting stability in order to prevent the forgetting of previous knowledge in a dynamically changing world. The nervous system solves this challenging problem in an unsupervised fashion and this problem has been referred to as the *stability-plasticity dilemma* (Grossberg, 1980, 2012).

This problem is further compounded in its complexity by the fact biological systems are open thermodynamic systems where energy and matter constantly flow through them (Katchalsky and Kedemo, 1962; Swenson and Turvey, 1991; Kello, 2013). This flow produces variations within the nervous system where action potentials are always generated by neurons such that synaptic strengths are constantly being modulated (Freeman, 2001) to adapt to a changing world, and network structures never stop changing (Pascual-Leone et al., 2005) and all these changes can happen at a variety of spatial and temporal scales.

In a well-known set of experiments by Freeman and Schneider (1982), rabbits were surgically implanted with a rectangular array of electrodes in the olfactory bulb. In one such experiment to test serial conditioning, odor stimuli in the form of sawdust, acetyl acetate, butyric acid and finally sawdust were presented serially to the rabbits. The neural activity in the bulb electrodes changed with each new odorant. On returning to the first odorant, the sawdust, neural activity was very different from those recorded on the first exposure. However, the rabbits exhibited repeatable behaviors such as avoiding odors that were undesirable while approaching toward other odors that were desirable. How is that the neural activity (or internal representations) in the brain can be so variable and yet the animal can produce stable and repeatable behaviors?

Neural models based on the adaptive resonance theory (Grossberg, 2012) attempt to answer these questions by using firing rate code combined with Hebbian plasticity models. Rate coding is based on the assumption that information is coded coarsely in the number of spikes occurring in a given window of time. The recently proposed *reservoir-computing model* (Maass et al., 2002; Buonamano and Maass, 2009; Maass, 2010) predicts that temporal integration of incoming information and generic non-linear mixing of this information within a liquid or recurrent network of excitatory and inhibitory neurons are primary computational functions of a cortical microcircuit. The state of the network at any given time can be represented by a point in high-dimensional space where each dimension corresponds to the activity level of a neuron. A temporal sequence of these points forms a neural trajectory. The advantage of computing with neural trajectories is that temporal information is implicitly encoded in them and can be read out by downstream neurons. This approach to computing has received experimental evidence (Hahnloser et al., 2002; Nikolic et al., 2009; Crowe et al., 2010; Long et al., 2010; Bernacchia et al., 2011; Klampfl et al., 2012). These models are based on firing rates of neurons (Jaegar and Haas, 2004; Sussillo and Abbott, 2009; Laje and Buonamano, 2013).

There is mounting evidence for temporal coding in the brain (Rieke et al., 1996; Victor and Purpura, 1996; Van Rullen et al., 2005; Dan and Poo, 2006; Tiesinga et al., 2008) where information is coded in the precise timing of individual spikes from individual neurons. The adaptive resonance models also do not consider spike frequency dependent short-term plasticity (Tsodyks and Markram, 1997; Tsodyks et al., 1998) and spike timing dependent long-term plasticity of excitatory and inhibitory synapses (Markram et al., 1997, 2011; Bi and Poo, 1998; Woodin et al., 2003; Vogels et al., 2011, 2013). Spiking versions of reservoir computing models have shown learning of spatiotemporal patterns (Maass et al., 2002; Maass, 2010) but the reservoir is not plastic in these implementations.

A spiking neural network with spike-driven synaptic dynamics compatible with STDP and short-term synaptic plasticity and with supervisory signals was shown to learn and correctly classify a large number of overlapping patterns (Brader et al., 2007). This network did not consider inhibitory synaptic plasticity dynamics and required plasticity to be turned off after learning. In a previous model, the authors showed that a similar supervisory signal driven spiking neural network learns spatiomotor transformations (Srinivasa and Cho, 2012). It was shown recently that incorporation of synaptic plasticity in the excitatory synapse and network motifs within a spiking reservoir can result in the emergence of long-term memory in the form of sequences of network states (Klampfl and Maass, 2013). However, this model does not have synaptic plasticity in both excitatory and inhibitory synapses. It also did not address the relation of their network to the unsupervised discrimination of patterns.

A spiking neural model with a reservoir type architecture is presented that is composed of a source layer with neurons that are activated by external inputs, a reservoir that resembles a generic cortical layer with an excitatory-inhibitory (EI) network and a sink layer of neurons for readouts. Synaptic plasticity in the form of STDP is imposed on all the excitatory and inhibitory synapses at all times. Using a novel discrimination measure called pattern discriminability index (*DI*), the spiking network is shown to be capable of discriminating between spatial patterns of spiking inputs in an unsupervised manner (i.e., without any explicit supervisory signals or labels) despite continuous synaptic plasticity.

The *DI* can be viewed a generalization of the average Hamming distance (Garcia-Sanchez and Huerta, 2004; Olypher et al., 2012) between neuronal patterns based on relative firing rate distributions. It also has close links to information theoretic measures (Borst and Theunissen, 1999) because it quantifies the amount of information the output neurons carry about the input patterns presented to the system during training.

## Materials and methods

### Model architecture

**Figure 1:**
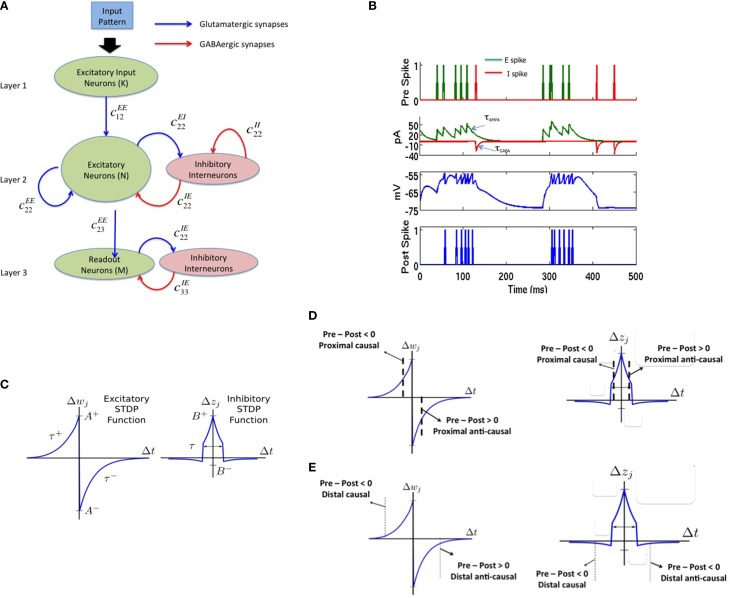
**The complete model with (A) a three layered network architecture with a source (layer 1), reservoir (layer 2), and sink (layer 3) neurons**. The source neurons receive inputs patterns in spike-encoded form. These spikes are then projected to the excitatory neurons in the reservoir layer that are recurrently connected to other neurons in the excitatory population. The excitatory population of neurons is also connected to an inhibitory population of neurons reciprocally. The inhibitory neurons are recurrently connected to neurons within its population. The connectivity between the various layers in the network are set as: *c*^*EE*^_12_ = 20%, *c^EE^*_22_ = 40%, *c^EI^*_22_ = 40%, *c^IE^*_22_ = 50%, *c^II^*_22_ = 50%, *c^EE^*_23_ = 30%, *c^EI^*_33_ = 100%, and *c^IE^*_33_ = 100% for all simulations. Here, *c^EI^*_22_ = 40% means that the connectivity between the *E* and *I* neurons in layer 2 is randomly connected at 40% of full connectivity between the two neuron populations. **(B)** The four subplots summarizes the leaky integrate and fire process in a typical neuron in our model. The first subplot shows input spikes from *E* (green) and *I* (red) pre-synaptic neurons. The second subplot shows the conversion of these spikes into currents that also includes the AMPA (green) and GABA (red) kinetics. The third subplot shows the integration of membrane voltage trace of the post-synaptic neuron based on the sum of the currents; and the last subplot shows the spikes generated by the post-synaptic neuron when the membrane voltage exceeds *V_T_*. **(C)** The E-STDP is an asymmetric function of the timing difference (Δ*t* = *t_pre_* − *t_post_*) between the pre- and post-synaptic spikes at neuron *j* and the corresponding change in synaptic conductance Δ*w_j_* for *E* → *E* and *E* → *I* synapses. The four parameters (*A*^+^, *A*^−^, τ^+^, τ^−^) control the shape of the function and thus the amount of potentiation and depression. The I-STDP is a symmetric function of the timing difference Δ*t* between the pre- and post-synaptic spikes at neuron *j* and the corresponding change in synaptic conductance Δ*z_j_* for *I → E* and *I* → *I* synapses. The three parameters (*B*^+^, *B*^−^, τ) control the shape of the function and thus the amount of potentiation and depression. **(D)** Inhibitory STDP interacts with excitatory STDP to favor balance among causal synaptic currents. Presynaptic and post-synaptic spikes can be proximal causal or proximal anti-causal to varying degrees. The dashed lines reflect an example of timing difference for which proximal causality is assumed. **(E)** Presynaptic and post-synaptic spikes can be distal causal or distal anti-causal to varying degrees. The dashed lines reflect an example of timing difference for which distal causality is assumed. The E-STDP and I-STDP combine to form four different interacting regimes: *Balance regime* that occurs for the proximal causal case, where excitatory and inhibitory conductance both increase; *Accelerated Potentiation regime* that occurs for the distal causal case, where excitatory conductance increases albeit by small amounts, while inhibitory conductance decreases by small amounts; *Decelerated Depression* regime that occurs in distal anti-causal case, where excitatory and inhibitory conductance both decrease by small amounts; and *Quiescent regime* that occurs in proximal anti-causal case, where excitatory conductance is strongly decreased and inhibitory conductance is strongly increased.

The spiking network model proposed in this paper consists of three layers as shown in Figure 1A. The source layer contains excitatory neurons that are stimulated by sources external to the network and projected to reservoir neurons. These projections were random and relatively sparse for the sake of simplicity. The reservoir neurons were either excitatory or inhibitory, received projections from source neurons and other reservoir neurons, and projected to other reservoir neurons and neurons in the third layer called the sink layer. The sink neurons received projections from the reservoir neurons but did not project back to the network. The sink neurons were composed of both excitatory and inhibitory neurons.

In this paper, the source layer (layer 1 in Figure 1A) contains *K* = 900 neurons (converted from a 30 × 30 2-D array into a linear array), the reservoir (layer 2 in Figure 1A) contains *N* = 200 excitatory and 50 inhibitory neurons (in a 4:1 ratio between excitatory and inhibitory neurons) and *M* = 8 excitatory neurons sink layer (layer 3 in Figure 1A) that are recurrently connected to inhibitory neurons in the sink layer. There are four types of synapses depending on the pre- and post-synaptic neuron type at each synapse: *E* → *E*, *E* → *I*, *I* → *E*, and *I* → *I*. The first two types of synapses are excitatory in nature and obey E-STDP rule while the last two types of synapses are inhibitory in nature and obey the I-STDP rule for plasticity. The connectivity between the layers in the network is set randomly with probability *c^AB^_ij_* where the superscripts A and B reflect excitatory (*E*) or inhibitory (*I*) type of neuron while subscripts *i* and *j* correspond to the sender and receiver layers (Figure 1A). All synapses are plastic throughout all simulations and synaptic connections are set randomly. The spiking model simulations were performed using the HRLSim (Minkovich et al., 2014) that is a multiple graphical processing unit (GPU) based spiking simulator in C++.

### Neuron model

The leaky integrate and fire neuron (Vogels et al., 2005) is used to model neuronal dynamics with a single compartment and no somatic, dendritic or axonal specialization. In response to multiple input currents coming from excitatory and inhibitory presynaptic neurons in the sets *Pre_ex_* and *Pre_inh_*, respectively, the membrane potential *V* for post-synaptic neuron *i* is determined by:

(1) $$\begin{array}{l} \tau_{m}\frac{dV_{i}}{dt}=\left(V_{rest}-V_{i}\right)+\left(E_{ex}-V_{i}\right)\sum_{j\in Pre_{ex}}g_{ex,\;ij}\\ \;+(E_{inh}-V_{i})\sum_{j\in Pre_{inh}}g_{inh,\;ij} \end{array}$$

When *V* reaches a threshold voltage *V_T_*, the neuron fires a spike (Figure 1B), and *V* is reset to *V_reset_*. The output information is encoded into the timing of these spikes. This basic model provides several control variables for the membrane voltage including conductances *g_ex_* (excitatory) and *g_inh_* (inhibitory), membrane time constant τ*_m_*, the constant reversal potential for excitatory (*E_ex_*), and inhibitory (*E_inh_*) synaptic currents, and a fixed voltage threshold for firing *V_T_* at which the neuron fires a spike. Synaptic inputs to the neuron are modeled as conductance changes where a set of excitatory or inhibitory presynaptic spike times, *S_ex_* or *S_inh_*, respectively, gives conductance dynamics:

(2) $$\frac{dg_{ex}}{dt}=-\frac{g_{ex}}{\tau_{AMPA}}+w\sum_{s\in S_{ex}}\delta (t-s)$$

(3) $$\frac{dg_{inh}}{dt}=-\frac{g_{inh}}{\tau_{GABA}}+z\sum_{s\in S_{inh}}\delta (t-s)$$

Here the time constants τ_*AMPA*_ and τ_*GABA*_ approximate the average decay of AMPA and GABA currents respectively (Figure 1B). The value of the excitatory and inhibitory synaptic conductance *w* and *z* is controlled by STDP (Figure 1C). In all simulations, τ*_m_* = 20 ms, *V_T_* = −54 mV, *V_rest_* = −74 mV, *V_reset_* = −60 mV, *E_ex_* = 0 mV, *E_inh_* = −80 mV, τ*_AMPA_* = 40 ms, and τ_*GABA*_ = 50 ms. All simulations used Euler integration with a time step of 1 ms (Srinivasa and Jiang, 2013).

### Excitatory STDP

The E-STDP function modulates the excitatory synaptic weight *w* based on the timing difference (*t_pre_*–*t_post_*), or Δ*t*, between the spike times of pre- and post-synaptic neuron (Figure 1C). The control parameters τ^+^ = 20 ms and τ^−^ = 20 ms determine the temporal window over which STDP is active. The change in synaptic weight is computed using the additive STDP rule as:

(4) $$w=w_{old}+\Delta w$$

(5) $$\text{where }\Delta \text{w}=\{\begin{array}{l} A^{+}exp^{\frac{\Delta t}{\tau^{+}}},\;△t<0\\ -A^{-}exp^{\frac{-△t}{\tau^{-}}},\;△t\geq 0 \end{array}$$

If *w_new_* > *g^E^_max_*, then *w_new_* = *g^E^_max_*. On the other hand if *w_new_* < 0, then *w_new_* = 0. The factors (*A*^+^, *A*^−^) correspond to the max synaptic change possible for potentiation and depression respectively at any given time step. The E-STDP parameters are set as: *A*^+^ = 0.005 nS and *g^E^_max_* = 0.3 nS. The factor β = |*A*^−^τ^−^|/|*A*^+^τ^+^| which controls the relative amounts of depression to potentiation during learning is set 1.05 that represents a slight bias toward depression (Song et al., 2000). The initial excitatory synaptic weight *w* was set by picking values randomly in the interval (0, 0.1 nS) for synapses in layers 1 and 2 and was set between (0, 0.2 nS) for synapses between layers 2 and 3.

### Inhibitory STDP

The I-STDP function modulates the inhibitory synaptic weight *z* (Vogels et al., 2011, 2013; Srinivasa and Jiang, 2013) based on the timing difference Δ*t* between the spike times of corresponding pre- and post-synaptic neurons (Figure 1C). The synaptic weight is computed as:

(6) $$z=z_{old}+△z$$

The change Δ*z* is governed by the following equations:

(7) $$\Delta z=\{\begin{array}{ll} B^{+}*\exp\left(\frac{-\left|\Delta t\right|}{\tau }\right), & if\;\left|\Delta t\right|\leq \tau \\ -B^{-}*\exp\left(\frac{-\left|\Delta t\right|}{\tau }\right), & if\;\left|\Delta t\right|>\tau \end{array}$$

If *z_new_* < 0 then *z_new_* = 0. On the other hand, if *z_new_* > *g^I^_max_* then *z_new_* = *g^I^_max_*. The I-STDP parameters are set as *B*^+^ = 0.0015 nS and *B*^−^ = 0.0003 nS, *g^I^_max_* = 0.2 nS and τ = 10 ms. The initial inhibitory synaptic weight set by picking values randomly in the interval (0, 0.1 nS) for all synapses.

### Interplay between E-STDP and I-STDP for balanced currents

Excitatory and inhibitory long-term plasticity are both important, as it is the interplay between these two effects that results in a network with a balance between excitatory and inhibitory currents at each neuron in the reservoir layer. The networks with such a current balance are referred to as *balanced networks* (Vogels et al., 2011; Srinivasa and Jiang, 2013). Networks without inhibitory STDP fail to reach this state for any of a large set of possible network parameters. Figure 1D shows a schematic description of how these two STDP functions combine to create a balanced network.

The inhibitory STDP function is symmetrical supporting an increase in synaptic conductance, i.e., synaptic inhibition, for closely timed pre- and post-synaptic spikes regardless of their order. In contrast, the excitatory STDP function is anti-symmetric and biased toward depressing action. Together, for each of these two STDP functions along the Δ*t* = *t_pre_* − *t_post_* timeline, there are four qualitative regions: proximal causal and anti-causal (Figure 1D), for those spikes that occur relatively close together, and distal causal and anti-causal (Figure 1E), for those that occur farther apart.

### Input image encoding and noise injection

**Figure 2:**
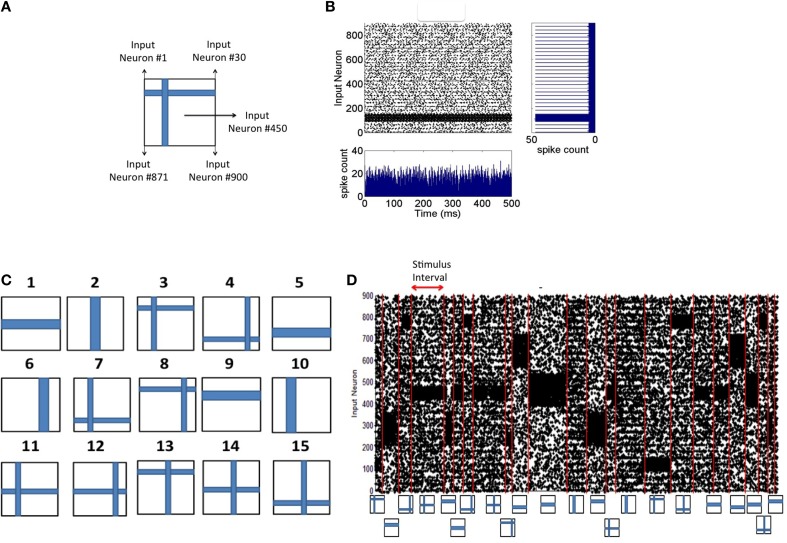
**Spike input encoding at the source layer of the network. (A)** An input pattern in the form of 2-D array of pixels is presented to the source neurons of the network that are linearly arranged. See Materials and Methods section for details of spike encoding. **(B)** The spikes generated during the presentation of the example in **(A)** for 500 ms duration is shown here. The firing rate at any given time (the bottom subplot) is approximately the same showing no undue bias introduced in the spikes that are fed to the source neurons. The linear arrangement of the source neurons results in the spike frequency plot (shown on the right) for the given input pattern computed for a duration of 500 ms. **(C)** Input patterns of the training set consists of *P* = 15 “flag” patterns where each pattern is a binary image array of 30 × 30 pixels. **(D)** An example sequence of input patterns after spike encoding at the source neurons is shown here. The duration of the presentation of each pattern varies and is chosen from an exponential distribution with a mean of 30 ms. Red lines demarcate the shift from one training pattern to another. The training patterns are selected from the set of 15 in a random order. The plot shows a total duration of 1800 ms with 10% of the source neurons injected with noise throughput the sequence.

Each 2-D input image pattern is first converted into a 1-D vector (Figure 2A). The 1-D input image vectors are then converted into spike sequences by an encoding process as follows. The neurons in the input layer are modeled using a Poisson process and each neuron receives an input from one pixel in the image. If a pixel is black in the input image, the neuron is assigned a mean firing rate of *f* = 90 Hz and if it is white, 10% of the source layer neurons with white pixels are assigned a mean firing rate of *f* = 10 Hz to simulate noise in the image. The spike encoding process is generated based on Poisson statistics. Assuming a sampling rate of *dt* and for a mean firing rate of *f* Hz for a given pixel, *f* spikes are generated every *1/dt* samples. Thus, the probability of spiking at each time step for a given pixel firing at *f* Hz is f^*^dt. Spike trains are generated for each pixel based on its probability of spiking at each source layer neuron. An example result of this encoding process for input patterns (Figure 2A) is shown in Figure 2B. In all simulations, *dt* = 1 ms as mentioned earlier.

### Input pattern presentation during training and testing

The training process consists of presenting each input pattern in the training set (Figure 2C) in a random order for a duration drawn from an exponential distribution with a mean of 30 ms (Figure 2D). The network is tested for discriminability at regular intervals (every 10 s) during which synaptic plasticity in the network is turned off. Each input pattern is presented during the testing process in a fixed sequence for *d* seconds each and the discriminability index is then computed based on the generated firing rate codes (as described below). The process of estimating *d* is also provided below.

### Firing rate code for readout neurons

**Figure 3:**
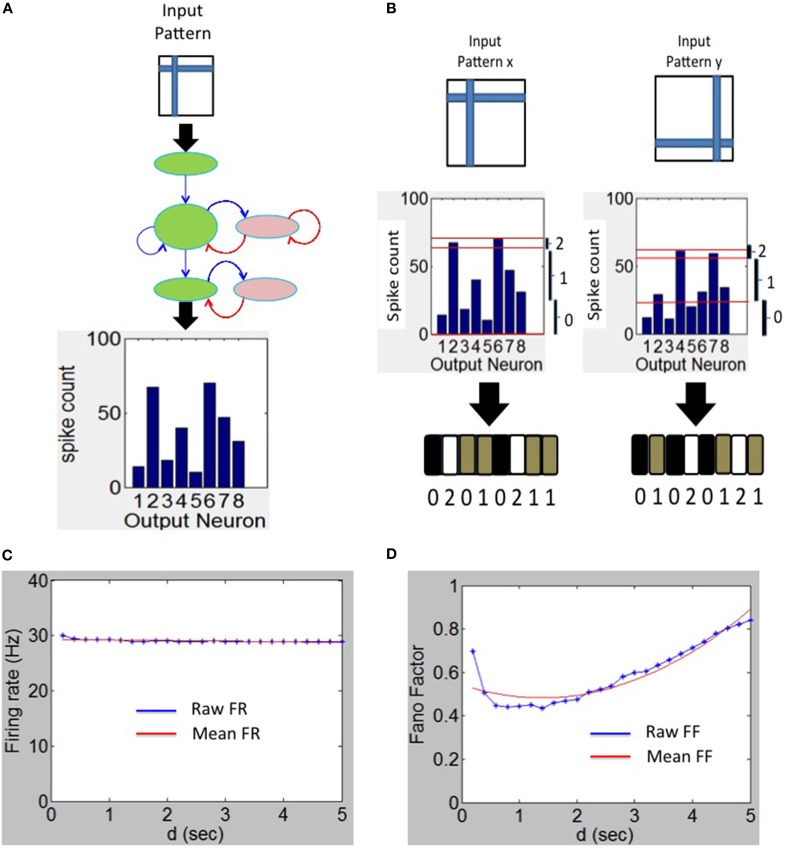
**Spike output decoding at the output layer of the network. (A)** The output spike histogram is shown for neurons at the sink layer (assumed to have 8 neurons in this example) for a given input pattern presented at the source neurons and **(B)** this histogram is converted to firing rate code *S^p^* with three possible states for each neuron (black—0 gray—1 and white—2). The firing rate code for two different input patterns results in two different codes as shown here. **(C)** The mean firing rate of the output neurons during the experiment to determine the duration *d* using the Fano Factor. **(D)** The variation of the Fano Factor as a function of the duration *d*.

The firing rate code for the readout neurons are evaluated only during the testing phase during which each input pattern from the training set is presented to the network for a duration of *d* seconds for a total duration of d^*^P seconds for *P* patterns. Each pixel in the input image stimulates one neuron in the source layer (Figure 3A). The source neurons are modeled as Poisson spike sources as described above. For each test pattern *p*, the the firing rates *f^p^_i_* of sink neuron *i* in layer 3 (Figure 3B) can be computed as the total number of spikes emitted during a duration of *d* seconds. The maximum firing rate *f^p^_max_* is then estimated from the firing rates of all sink neurons for that test pattern *p*. The firing rate vector *S^p^* of length *M* for pattern *p* is composed of components *S^p^_i_* for each sink layer neuron *i* can be computed as:

(8) $$S_{i}^{p}=\{\begin{array}{ll} 2, & \;if0.9\leq \frac{f_{i}^{p}}{f_{aax}^{p}}<1.0\\ 1, & \;if0.4\leq \frac{f_{i}^{p}}{f_{aax}^{p}}<0.9\\ 0, & if\frac{f_{i}^{p}}{f_{aax}^{p}}<0.4 \end{array}$$

The vector *S^p^* is referred to as the firing rate code and in this example it is a *tertiary* firing rate code (i.e., *C* = 3) because each sink neuron can have three possible states for a given input pattern *p*. An example of this tertiary code for two different input patterns is shown in Figure 3C. It is possible to use other coding levels such as binary (*C* = 2) or quarternary (*C* = 4) codes. In this paper, *C* = 3 is used as it offers the highest discriminability as explained in the Results section.

### Estimating *d* for testing

The firing rate code S (above) and the estimation of discriminability index (as explained below) depends upon the duration *d* of each test pattern presentation. To estimate an appropriate duration *d*, the Fano factor (Churchland et al., 2010; Eden and Kramer, 2010) was computed from the spikes generated by the readout neurons by assessing the relationship between variability of the spike counts and duration *d*.

The Fano factor (*FF*) is defined as the ratio of sample variance to sample mean of spike counts observed in a time window and the quality of the estimator strongly depends on the length of the window. The *FF* measures the noise-to-signal ratio and therefore characterizes the neural variability over trials. For example, for a Poisson process, the variance equals the mean spike count for any length of the time window. If the *FF* has a minimum at some value of *d*, this can be an optimal value for *d* since the firing rate code would be robust at that value (see e.g., Ratnam and Nelson, 2000; Chacron et al., 2001).

The *FF* was computed for various durations *d* as follows. The spikes for each test pattern presented for duration of a selected *d* was first collected for each of the *M* readout neurons separately. This was repeated for 100 trials to collect a set of M^*^100 spike count values. The mean and variance in the spike count was then computed from these values. The ratio of the computed variance to mean gives *FF* for the selected *d* and for the selected test pattern. This process was repeated for all remaining *P*-*1* test patterns and the resulting average *FF* was used as the *FF* for a given duration *d*. Since the average firing rate of the sink layer neurons was steady between 28 and 30 Hz (Figure 3C), the mean-matching procedure for *FF* (Churchland et al., 2010) was not used. To estimate the appropriate duration *d*, the *FF* was plotted as a function of duration *d* (Figure 3D) for *M* = 8 and *P* = 15. The minimum *FF* is ~0.48 and occurs at *d* = 1.4 s. We set *d* = 1.4 s in all our simulations.

### Discriminability index computation

During the learning process, as input patterns are presented, a firing rate code *S^p^* can be computed at the sink layer for each pattern *p* presented to the source layer as described above. The ternary firing rate code changes as the network is presented with more inputs. This implies that the ternary code cannot be directly used for reliably separating one pattern from another. However, after a few pattern presentations, the ability of the network to discriminate between the patterns becomes stable and reliable.

To verify this, a discriminability index (*DI*) was computed as follows. At regular intervals (once every 10 s) the network was stopped to probe the state of the network. During this process, the synaptic weights are frozen and each pattern is presented *J* times for duration of *d* = 1.4 s each. For a given pattern *p*, the firing rate code *S^p^* was computed for each of the *J* presentations of *p*. A *prototype firing rate code* was selected for a given pattern *p* as the code that is most repeating among the *J* codes generated. If there are no repeats, one of the *J* codes as the prototype was selected at random. This process is repeated for each pattern to identify a prototype firing rate code for each input pattern. Using the prototype firing rate codes, the *inter-pattern distance* (*D_inter,_*) was computed. *D_inter_* is defined as the average pair-wise distance between prototype readout codes computed from all possible unique pairs of prototype readout codes generated by the network for a given test set. To calculate *D_inter_*, the distance *d^pq^_i_* between a pair of *S* codes for two input patterns *p* and *q* and for each sink neuron *i* was computed as:

(9) $$d_{i}^{pq}=\{\begin{array}{ll} 0 & if\;S_{i}^{p}=S_{i}^{q}\\ 1 & if\;S_{i}^{p}\neq S_{i}^{q} \end{array}$$

The distance *D_inter,i_* was then computed by using *d^pq^_i_* for every pair of input patterns *p* and *q* for each sink neuron *i* across all test patterns *P* as:

(10) $$D_{inter,\;i}=\frac{\sum_{k=1}^{P-1}\sum_{j=k+1}^{P}\sum_{i=1}^{M}d_{i}^{kj}}{P*(P-1)/2}$$

The maximum value of *D_inter,i_* for a readout code can be estimated as follows. Assuming a ternary readout code at the sink layer (i.e., *C* = 3 and that *P* is odd, the maximum pairwise distance between the readout code at each sink layer neuron *i* is obtained when the readout is equiprobable with “0” for one third of *P* input patterns, “1” for another third of inputs and with “2” for the remaining third. The theoretical maximum value of the numerator Equation (10) can be computed as P^*^P/3 and thus *D^max^_inter_* can be computed as 2*P*/(3^*^(*P* − 1)). If *P* is even, *D^max^_inter_* can be similarly computed as 2(*P* + 1)/3^*^P. Similarly, for a binary code (e.g., *C* = 2) *D^max^_inter_* can be computed to be (*P* + 1)/(2^*^P) when *P* is odd and 2*P*/(3^*^(*P* − 1)) when *P* is even. Thus, *D^max^_inter,i_* can be computed for the general case when *C* is even as:

(11) $$D_{inter,i}^{max}=\{\begin{array}{l} \frac{P(C-1)}{C\left(P-1\right)}\;if\;P\;is\;even\\ \frac{\left(P+1\right)(C-1)}{CP}\;if\;P\;is\;odd \end{array}$$

Similarly *D^max^_inter,i_* for the general case when *C* is odd can be expressed as:

(12) $$D_{inter,i}^{max}=\{\begin{array}{l} \frac{P(C-1)}{C\left(P-1\right)}\;if\;P\;is\;odd\\ \frac{\left(P+1\right)\left(C-1\right)}{CP}\;if\;P\;is\;even \end{array}$$

The expression for the inter-pattern distance *D_inter_* can be written in terms of *D_inter,i_* as:

(13) $$D_{inter}=\sum_{i=1}^{M}D_{inter,\;i}$$

By substituting *D^max^_inter,i_* from Equations (11) or (12) (depending upon whether *C* are even or odd respectively) into Equation (13), the theoretical maximum value of *D_inter_* can be computed. For example, if *C* is even, *D_inter_* will be *PM(C-1)/(C(P-1))* if *P* is odd and *(P+1)M(C-1)/CP* is *P* is even. Thus, if *M* = 8, *C* = 2 and *P* = 15, the theoretical maximum for *D_inter_* will be 4.28. It should also be noted that the theoretical maximum for *D_inter_* grows linearly with *M*. The theoretical maximum for *D_inter_* will serve as the upper bound for a given set of parameters during learning. This is because there is noise in the network that prevents an equiprobable distribution of readout codes by the network.

An *intra-pattern distance* (*D_intra,_*) was also computed by presenting the same pattern *J* times for *d* seconds each. *D_intra_* is defined as the average pair-wise distance between readout codes same as Equation (10) computed from all possible unique pairs of readout codes generated by the network for the same input pattern. This distance provides a measure of an average variation in the response of readout neurons for the same input pattern. This variation can be caused due to noise in the inputs. It should be noted that *J* = 10 in all our simulations.

The discriminability index (*DI*) is then defined as a product of two measures. The first is called *separability*, ε, that measures the degree of separation of readout codes for a given test set. This measure can be computed as:

(14) $$\varepsilon =1-\frac{D_{intra}}{D_{inter}}$$

This measure is akin to computing the Fischer metric (McLachlan, 2004). A small *D_intra_* relative to *D_inter_* implies that the network can separate the inputs well. Separability is independent of *M*.

The second measure is called the *uniqueness*, *γ*, that is defined as the number of unique readout codes produced by the network relative to maximum possible number of unique readout code. This can be expressed as:

(15) $$\gamma =\frac{\#S}{P}$$

where #S refers to the total number of unique readout codes for a given test set of size *P*. Uniqueness is dependent on *M* since high dimensional readout codes generate more unique codes (Kanerva, 1988). The discriminability index (*DI*) is then computed as:

(16) DI=ε ∗ γ

High values of *DI* correspond to readout codes that are have a low *D_intra_* combined with high *D_inter_* or high separability as well as a high uniqueness. The maximum value of *DI* is 1.0 and its minimum value is typically zero unless *D_intra_* > *D_inter_*. *DI* is dependent on *M* since uniqueness is dependent on *M* (see Appendix for an example calculation of *DI*).

### Synaptic distance computation

In order to analyze for stability of learned codes, the *synaptic distance* was computed to track the synaptic changes between layers of the network for excitatory synapses. Since the E-STDP plasticity rule used in this paper is of the additive type, the resulting distribution of synapses after learning is bimodal in nature (Song et al., 2000). This bimodal distribution is due to competition that occurs among synapses at each neuron. The synapses that cause the post-synaptic neuron to fire more frequently will potentiate to the maximum synaptic weight while the other uncorrelated synapses will depress to a zero. To calculate the synaptic distance, the synaptic weights *w_ij_* are converted into a binary weight *W_ij_* where *W_ij_* = 1 if (i.e., *w_ij_* > 0.7^*^g^E^_max_) and *W_ij_* = 0 otherwise. The synaptic distance ϕ_*kl*_(*t*_1_, *t*_2_) between excitatory synapses from layer *k* to layer *l* at time *t*_1_ with the same synapses at time *t*_2_ can be expressed as:

(17) $$\phi_{kl}(t_{1},t_{2})=\frac{\sum_{j=1}^{\#l}\sum_{i=1}^{\#k}\left|W_{ij}\left(t_{2}\right)-W_{ij}(t_{1})\right|}{\#k*\#l}$$

where #*k* and #*l* correspond to the number of neurons in layer *k* and *l* respectively and binary *W_ij_(t)* corresponds to the *ith* synapse in the *kth* layer that is connected to the *jth* synapse in layer *l* at time *t*. For example, #*k = K* for layer 1 and #*l* = *N* for layer 2 in the network. The synaptic distance is the total Hamming distance between the binary weights at two different time steps (*t*_1_, *t*_2_) where *t*_2_ > *t*_1_.

In addition to computing the synaptic distance, a shuffled synaptic distance was computed as a control to compare the synaptic weight changes during learning to those that could arise from chance. This distance ϕ*^shuffled^_kl_*(*t*_1_, *t*_2_) between excitatory synapses from layer *k* to layer *l* at time *t*_1_ with the same synapses at time *t*_2_ can be expressed as:

(18) $$\phi_{kl}^{shuffled}(t_{1},t_{2})=\frac{\sum_{k=1}^{\#shuffles}\sum_{j=1}^{\#l}\sum_{i=1}^{\#k}\left|\begin{array}{c} W_{ij}^{shuffled}\left(t_{2}\right)\\ -W_{ij}(t_{1}) \end{array}\right|}{\#shuffles\;*\;\#k\;*\;\#l}$$

where #shuffles is the total number of shuffles that *W^shuffled^_ij_*(*t*_2_) undergoes at time *t_2_*. In all simulations, #shuffles = 10. By combining the above two measures, a relative synaptic distance measure ϕ*^rel^_kl_*(*t*_1_, *t*_2_) can be expressed as:

(19) $$\phi_{kl}^{rel}\left(t_{1},\;t_{2}\right)\;=\frac{\;\left|\phi \left(t_{1},t_{2}\right)-\phi^{shuffled}(t_{1},\;t_{2})\right|}{\phi^{shuffled}(t_{1},\;t_{2})}$$

If ϕ*^rel^_kl_*(*t*_1_, *t*_2_) is closer to 1.0, then ϕ*_kl_*(*t*_1_, *t*_2_) ≪ ϕ*^shuffled^_kl_*(*t*_1_, *t*_2_) and that implies that the distance between the synaptic weights at time *t*_1_ and *t*_2_ is very small compared to chance. This implies that the learning has stabilized in the network.

## Results

An initial training set was constructed composed of *P* = 15 flag patterns (Figure 2C). The patterns are presented in random order for a duration selected from an exponential distribution with a mean of 30 ms. Each pattern generates a Poisson spike train (Figure 2D) at the source neurons (see Materials and Methods). These spikes generated by each pattern in the input layer are transmitted to the *E* reservoir neurons in the middle layer for further processing.

### Balance of excitation and inhibition during learning of receptive fields

**Figure 4:**
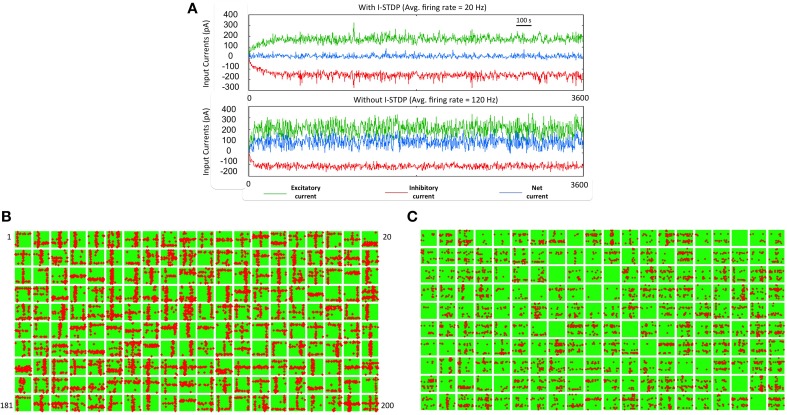
**Robust learning of receptive fields requires a balanced current regime. (A)** The sub-plot on top shows the total excitatory (green), and inhibitory (red) currents in the reservoir with I-STDP turned on. The net current (blue) is close to zero with several minor transients from zero. The average firing rate of the network is 20 Hz. If the I-STDP is turned off and with fixed inhibitory synaptic conductance, as shown in the second sub-plot, the total excitatory current is much higher than the total inhibitory current resulting in an unbalanced network with a very high average firing rate of 120 Hz. It may be possible to find a suitable synaptic conductance to achieve a balanced network but the use of I-STDP enables a self-organized process for achieving the balance. **(B)** The synapses between source neurons and the *E* neurons of the reservoir form a bimodal distribution where most of the synapses are weak with a few strong synapses. This process makes the connectivity between source and reservoir sparse compared its initial connectivity of 40%. Each box shows the synapses in a 30 × 30 image format from the source neurons to each of the 200 *E* neurons in the reservoir. The red dots within each box correspond to synapses that are greater than 0.7^*^g_max_ while the rest are shown as green dots. Each box is a receptive field of an *E* neuron in the reservoir and the set of all boxes forms a basis set for the training set. **(C)** The receptive fields with I-STDP turned off are not well-defined as with I-STDP being on. Many receptive fields are not formed at all while many others are have features that do not reflect any structure found in the input patterns.

As the patterns are presented, STDP in both excitatory (*w*) and inhibitory (*z*) synapses helps to achieve a good balance of excitation and inhibition currents in the network (Figure 4A). The synaptic weights *w* strengthens and creates an imbalance in synaptic currents due to inputs from the source neurons. At the same time the synaptic weights *z* gets rapidly potentiated due to I-STDP where inhibition increases irrespective of the order of occurrence of pre- and post-synaptic spikes for small timing differences between pre- and post-synaptic spikes. This results in a rapid compensatory increase in inhibitory currents into the neurons effectively preventing the neurons from exceeding *V_T_* more often (Vogels et al., 2011; Srinivasa and Jiang, 2013). Thus, the network is provided with very brief windows of opportunity to learn. These small windows of opportunity correspond to the transients in the balanced current (i.e., brief excursions of net currents above zero as shown by the top plot of Figure 4A). The excitation due to the input pattern is sufficient to overcome inhibition momentarily before inhibition and its modulation via inhibitory plasticity compensates for any discrepancy in the current balance.

As the training patterns are presented under the balanced current regime, synapses between the source layer neurons and each *E* neurons of the reservoir in layer 2 collectively form receptive fields (Song et al., 2000; Srinivasa and Jiang, 2013). This is achieved by adjusting the strength of these synapses via E-STDP. The red dots within a box (in Figure 4B) represent strong synapses between source neurons and an *E* neuron in the reservoir after 1 h of training. This process of synaptic strengthening is incremental and occurs using aggregates of input samples.

When the excitatory synapses alone obeyed the STDP rule and the inhibitory synapses were fixed (i.e., *z* = const) the excitatory and inhibitory currents are not balanced anymore (Figure 4A). There were also many *E* neurons that had no strong synapses. The strong synapses that emerge within each box (Figure 4B) appear to have a vertical or horizontal stripe (or both) and resemble the features of the input patterns in the training set. The learning of the receptive fields is also influenced by recurrent connections (i.e., *E* → *E*, and *I* → *I*) as well as mutual connections between the *E* and *I* populations (i.e., *E* → *I*, *I* → *E*) within the reservoir. All excitatory and inhibitory synapses within the reservoir are modified by E-STDP and I-STDP respectively. In order to assess the effect of turning off I-STDP, the receptive fields were analyzed after 1 h of training. Here the inhibitory weights z were randomly initialized between 0 and 1 but fixed throughout the simulations. The receptive fields did not have large variations in connectivity compared to the case where I-STDP was on Figure 4C.

**Figure 5:**
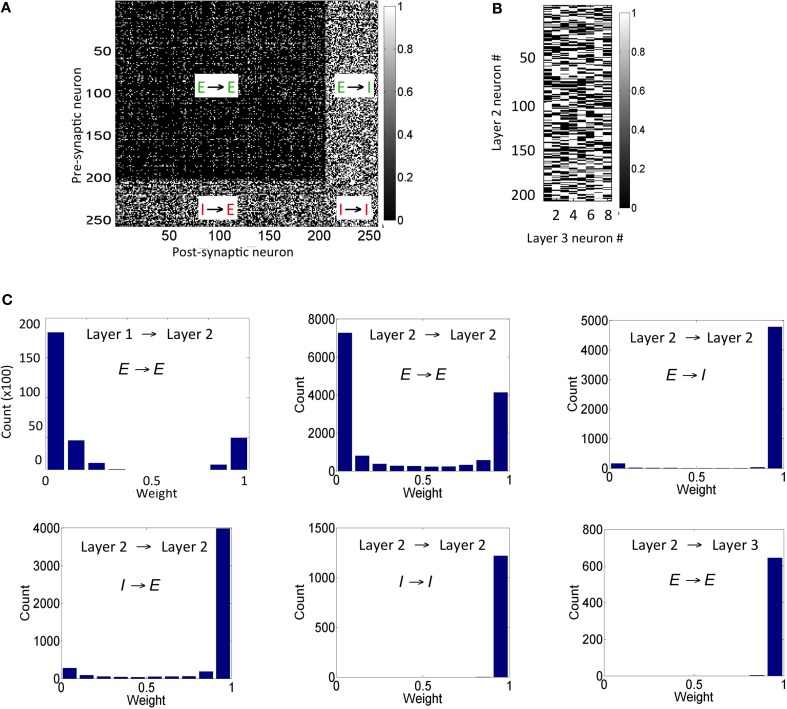
**Synaptic states for the network after 1 h of training are shown here. (A)** The synaptic weights within the reservoir are shown here. **(B)** The synapses between layer 2 and layer 3 are shown here. **(C)** The distribution of synapses between the *E* neurons in layer 1 and layer 2 as well as between *E* neurons within layer 2 is bimodal in nature. The distribution of synapses between *E* and *I* neurons with other *I* neurons within layer 2 is unimodal. The distribution for synapses between *E* neurons in layer 2 and *E* neurons in the layer 3 is also unimodal (see text for details). It should be noted that the weights in these plots are normalized with respect to *g^E^_max_* and *g^I^_max_* as appropriate.

The connection strengths for synapses in the reservoir after the presentation of the training set for duration of 1 h shows that the synapses between any inhibitory pre-synaptic neuron and either *E* or *I* post-synaptic neuron are mostly strong (Figure 5A) and the synaptic strengths are distributed in a unimodal fashion (Figure 5C). However, the *E* → *E* synapses between layer 1 and layer 2 are sparse with few strong synapses (~5% of all synapses). This discrepancy is primarily because E-STDP is anti-symmetrical while I-STDP is symmetrical. In other words, E-STDP is order dependent while I-STDP is not. Thus, I-STDP can potentiate synapses for both proximal causal and anti-causal spikes (Figure 1D). However, E-STDP will only potentiate the synapses if the pre-synaptic spike is causal to the post-synaptic spike. This implies that probability of potentiation is much higher for inhibition compared to excitation thus resulting in a bimodal distribution of synaptic strengths with many strong inhibitory synapses.

The synapses between the *E* neurons in the reservoir and the *E* neurons in the sink layer are also modified due to E-STDP (Figure 5B). The *E* neurons in the sink layer serve as *readout* neurons (Buonamano and Maass, 2009; Buzsáki, 2010). The distribution of synaptic strengths is unimodal (Figure 5C) unlike other *E* → *E* synapses described above. This is because of the following reason. Initially the strength of the synapses between the reservoir and the readout neurons is small (i.e., between 0 and 0.2). The connectivity between them is also sparse (i.e., *c^EE^*_23_ = 40%). Any given input at the source neurons causes a sequence of spiking activity in the reservoir neurons that is signaled to the readout neurons. This sequence of spiking activity among the reservoir neurons is hereinafter referred to as a *neural trajectory*.

Since the readout neurons are driven to fire by the reservoir neurons and by no other means, the temporal causality for their spiking is always from the reservoir neuron to the readout neuron. This results in the strengthening of synapses allocated to a readout unit (due to E-STDP). As the readout neurons fire in response to neural trajectories in the reservoir, all the synapses from the reservoir neurons to the readout neurons strengthen to its max value resulting in a unimodal distribution (Figure 5C). This is unlike the interaction between the source and reservoir neurons or within reservoir neurons where the spiking activity is driven by both feed-forward and lateral connections.

### Learning to discriminate patterns

**Figure 6:**
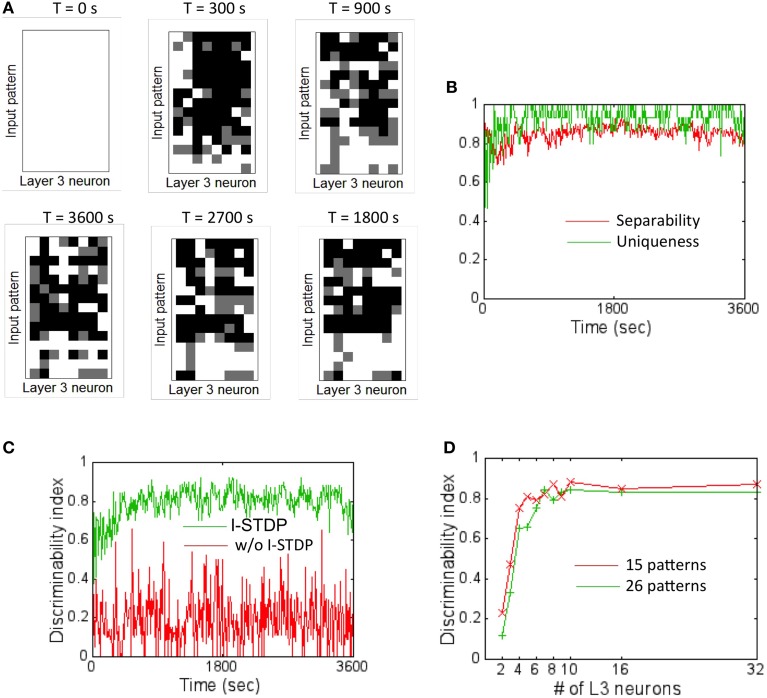
**Firing rate codes and *DI* computation. (A)** The firing rate codes were extracted from the spiking activity of the readout neurons (assuming 8 readout neurons). These codes were compared between input patterns at regular intervals during the training process. The inter-pattern readout codes are constantly changing (compare top row of readout code corresponding to input pattern #1) suggesting that the discrimination is very poor if readout codes are compared in an absolute fashion. **(B)** A plot of separability and uniqueness generated during the first hour of training with 15 input patterns is shown here for the case with I-STDP turned on. The two metrics are consistently high implying that the readout codes are highly separable as well as very unique. **(C)** A plot of *DI* for the case when I-STDP is turned on (green) compared to when it is turned off (red). This *DI* is computed by multiplying the separability with uniqueness (see text for details). **(D)** The *DI* is plotted against various number of readout neurons in the sink layer to assess the minimum number of readout neurons required to achieve high discriminability for two different sizes of training sets. While it is possible to achieve a *DI* ~0.8 for the 15 pattern case with only 5 readout neurons, higher number of patterns require more readout neurons. Since the total number of input patterns to be tested is 26 in this paper, a total of 8 readout neurons (or *M* = 8) was assumed for all simulations.

In order to assess the pattern discrimination capability, the training set was presented to the network for a total of 3600 s (see Materials and Methods). The firing rate of the readout neurons was monitored after every 10 s of training to test the network's ability to discriminate the input patterns. At these time intervals, plasticity was turned off and each input pattern was presented in a random order for 5 s and the *DI* metric Equation (16) was computed based on the activity of the readout neurons. The ternary code from the readout codes was plotted for each input pattern at regular intervals during the course of training (Figure 6A). The readout code initially looks alike for all patterns since the network has not really been exposed to all the patterns. As the network is exposed to more training data, the readout codes begin to show more variations. However, when the readout codes for an input pattern at two different times are compared, they appear to change constantly throughout the duration of the training period. This implies that a static template based discrimination algorithm would not be appropriate here.

The separability and uniqueness were tracked during the first hour of training (Figure 6B). Since the receptive fields form early due to E-STDP in the balanced regime created by the regulatory actions of I-STDP, good separability Equation (14) and uniqueness Equation (15) occur early during the training process. The *DI* Equation (16) thus rapidly rises to ~0.8 (Figure 6C) implying very good discriminability. The *DI* is however highly unstable and averages to ~0.2 when I-STDP is turned off. This is because the average firing rate of the network reaches 120 Hz (Figure 4A). This high firing rate results in poorly formed receptive fields (Figure 5B). This in turn results in very unstable separability and low uniqueness (not shown). Thus, the discriminability is poor and unstable (Figure 6C) when I-STDP is turned off.

The network size is potentially large enough to learn to discriminate many more patterns than used in the training set. However, the number of readout neurons was limited to the minimum required for obtaining good discriminability. To determine the minimum number of readout neurons for the chosen size of training set, the *DI* was averaged across 10 trials (Figure 6D) for two different sizes of training set: one with 15 patterns and the other with 26 patterns. While for the 16 pattern case, the *DI* rises to a value close to 0.8 with just four readout neurons, the network requires eight readout neurons to produce an average *DI* of ~0.8.

**Figure 7:**
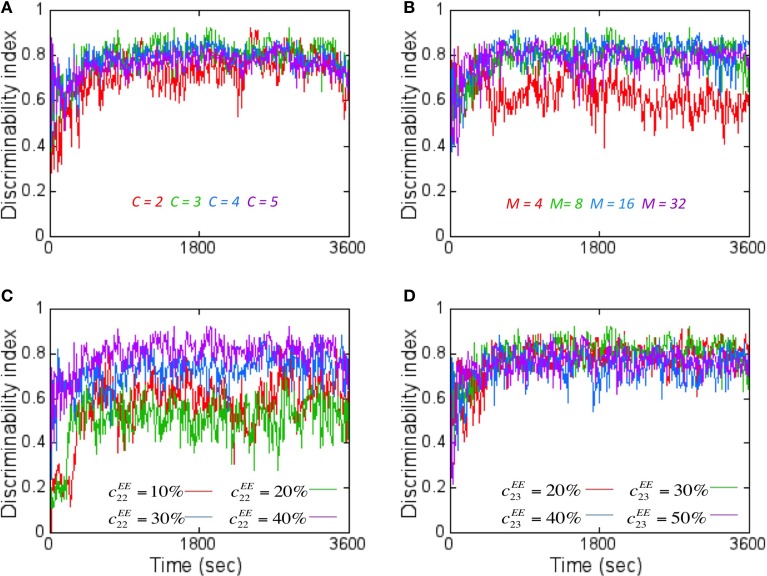
**The effect of various network parameters on *DI* is shown here. (A)** The coding level *C* for the readout code was varied to assess its effects on *DI* assuming *M* = 8. A coding level *C* = 2 (red) corresponds to just two states (On or Off) for the readout neurons while tertiary (green), quaternary (blue), and quinary (purple) codes correspond to 3–5 states respectively. *C* = 3 (green) produced the best average *DI* score. **(B)** Increasing the number of readout neurons *M* affects the *DI*. The worst average *DI* was for *M* = 4 while the best average *DI* was close for *M* = 8, 16, and 32. *M* = 8 was chosen for reasons mentioned in Figure 6. Here a ternary code (*C* = 3) is assumed. **(C)** The effect of increasing the lateral connectivity within the reservoir neurons does improve the *DI* metric but the degree of improvement starts to diminish with connectivity beyond 40%. Here *M* = 8 and *C* = 3 is assumed. **(D)** The effect of increasing the connectivity between *E* neurons from layer 2 to layer 3 results in only a marginal improvement in *DI* values. Here *M* = 8 and *C* = 3 is assumed.

When *DI* is computed using codes other than the ternary code (see Materials and Methods), it was worse (Figure 7A). This was unexpected since the number of possible states for the readout neuron should grow (i.e., 3^8^ states for ternary code vs. 5^8^ for quinary code) with number of states in the code. The main reason for this unexpected result is because *D_intra_* also grew at a faster rate with higher code values compared to *D_inter_* that grew at a slower rate with higher code. Thus, the separability reduces with higher code thus reducing *DI*.

When the number of readout neurons *M* was increased, the *DI* does change (Figure 7B). The effect of connectivity on *DI* was studied by tracking *E* → *E* connections within the reservoir in layer 2 as well as between layer 2 and layer 3. Adding more connections within the reservoir (i.e., *c^EE^*_22_) improved *DI* (Figure 7C). Similarly, on average, the *DI* improved when the number of connections between layer 2 and layer 3 (i.e., *c^EE^*)_23_ is increased (Figure 7D). All simulations primarily used *C* = 3, *M* = 8 and with *c^EE^*_22_ = 40% and *c^EE^*_23_ = 30%.

### Importance of learned connectivity and firing rate code for discrimination

In order to assess the effect of learned connectivity due to STDP on the discrimination ability of the network, a set of control experiments were performed. During each testing step (at a sampling interval of 10 s) the connectivity between three layers of the network was shuffled in six different ways while maintaining both the synaptic strengths and the total number of synaptic connections intact compared to the network with learned connectivity (or the original network). In the first case, shuffling was performed between connections between the source layer and the *E* neurons of the reservoir only. This was accomplished by randomly assigning pre-synaptic neurons from the source layer to post-synaptic *E* neurons in the reservoir that were different from the learned connections. In the second case, the learned connections between neurons within the reservoir (irrespective of whether they were *E* or *I* neurons) were randomly shuffled. In the third case, only the connections from *I* neurons in reservoir to all other connections (irrespective of whether they were *E* or *I* neurons) were randomly shuffled. In the fourth case, only the connections from *E* neurons in reservoir to all other connections (irrespective of whether they were *E* or *I* neurons) were randomly shuffled. In the fifth case the connections between the *E* neurons in the reservoir and the *E* neurons in the output layer were randomly shuffled. In the final case, shuffling was performed between all the layers—that is a mixture of shuffling performed for first through fifth cases.

**Figure 8:**
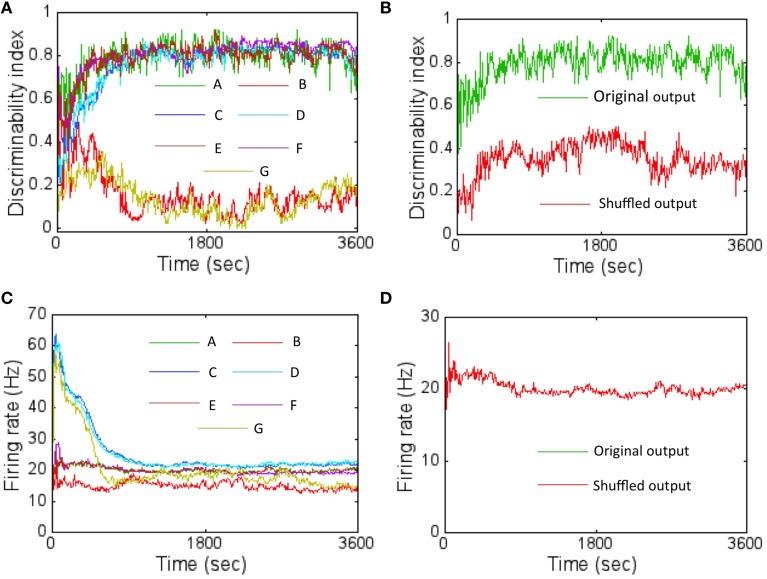
**The DI was computed with shuffled network connectivity to assess the importance of the learned connectivity for pattern discrimination. (A)** The results from the six control experiments (B: layer 1 → 2 synapses shuffled shown in red; C: layer 2 (*E* or *I*) to layer 2 (*E* or *I*) shown in blue; D—layer 2 (*I*) to layer 2 (*E* or *I*) shown in cyan; E—layer 2 (*E*) to layer 2 (*E* or *I*) shown in brown; F—layer 2 (*E*) to layer 3 (*E*) shown in magenta; G—all these connections shuffled simultaneously) are compared against the original network with learned connectivity **(A)** shown in green. The DI scores represent an average obtained after shuffling 10 times for each control experiment. The plot shows that altering the connectivity by shuffling the connections between layer 1 to *E* neurons in layer 2 affects discriminability severely while shuffling connections within layer 2 does not (see text for more details). **(B)** A control experiment was also performed to assess the importance of the specific locations of the firing activity within the readout code. This was achieved by first randomly shuffling the components of the readout code 10 different times. The *DI* scores were then computed for each case and then averaged. The results shows that the *DI* score is much lower (red trace) compared to the original network (green trace) suggesting that the locations of the firing activity within the readout code caused due to learning is also very important. **(C)** The average firing rate of the network for the various control experiments in **(A)** is shown here. **(D)** The average firing rate for the control experiment in **(B)** is shown here. The two firing rates overlap completely (red and green overlap completely). This is because there is no change in the connections between layer 2 and layer 3 neurons after STDP potentiates all of them over time early within the first hour. Furthermore, all connections go to its max value (i.e., unimodal distribution). So, swapping the outputs does not make a difference to the firing rates.

For each of these cases, the *DI* was computed (after every 10 s for a period of 1 h) by averaging the score after random shuffling 10 times in each case. The *DI* was worse for the first case compared to the original network (Figure 8A). In this case, swapping the connections between the *E* neurons in the source layer and the *E* neurons in the reservoir results in disturbing the learned receptive fields. This implies that the learned receptive fields between the source layer *E* neurons and the *E* neurons in the reservoir are very important for pattern discrimination in this network. However, interestingly, the learned connections between the neurons in the reservoir (the second through fourth cases) or the connections between *E* neurons in the reservoir and the *E* neurons in layer 3 (the fifth case) did not have a major effect in the *DI* scores.

For the second through fourth cases, the effect of shuffling the connections within the reservoir did not affect the *DI* score very much irrespective of whether the connections were from *E* or *I* neurons. The lateral connectivity between *I* → *I* or between *I* → *E* neurons only serves to regulate the balance of currents. Furthermore, all the synapses from *I* neurons are fully potentiated (i.e., unimodal distribution as shown in Figure 5C). Similarly, the most of the connections from *E* → *I* neurons are also fully potentiated (Figure 5C) while most of the *E* → *E* connections are weak (bimodal distribution as shown in Figure 5C). So swapping connections between strong *I* connections (for the third case) or between weak *E* → *E* or between strong *E* → *I* connections (for the fourth case) does not seem to change the network performance much as well. Since the second case is a combination of the third and fourth cases, the result is similar. For the fifth case, the connections between *E* neurons in the reservoir and the *E* neurons in layer 3 become fully potentiated (Figure 5C). So, swapping connections between strong *E* connections does not affect the *DI* score. Since the sixth case includes the first case as a subset, the *DI* score is severely affected much like in the first case.

The average firing rate of the network was initially most affected for the third and sixth control experiments while the first control experiment reduced the average firing rate of the network (Figure 8C) relative to the original network. For the third case, initially swapping the connections between *I* → *I* or between *I* → *E* affects the current balance as the synapses have not had the time to fully potentiate to its peak values. This in turn can affect the firing rates by causing them to be high. However, as learning proceeds, the balance is restored due to I-STDP causing these inhibitory synapses to become fully potentiated (Figure 5C) and the firing rates fall as expected (Figure 8C). Since the sixth case contains the third as a subset, it follows the same trend as the third. For the first case, the firing rates fall below the original network because swapping the connections between the *E* neurons in the source layer and the *E* neurons in the reservoir results in disturbing the learned receptive fields. Thus, on average, the *E* neurons in the reservoir do not have good matches with the input patterns because of the shuffling resulting in lower average firing rates.

In order to assess the importance of location of the active nodes in the firing rate code on the discrimination ability of the network, another control experiment was performed. The firing rate code was shuffled by shifting the location of the active nodes in the sink layer and this was repeated 10 times. Once every 10 s, the *DI* was computed with the shuffled firing rate code and then averaged to produce a *DI* that was compared against the *DI* generated by the original network. The shuffling of the active nodes results in much lower *DI* compared to the original network (Figure 8B). The firing rate of the network is unaltered by shuffling (Figure 8D) because the connectivity from *E* neurons in layer 2 to the *E* neurons in layer 3 has a unimodal distribution (Figure 5C) with all synapses being fully potentiated and thus being immune to the shuffling.

### Stability of learning

The network was analyzed for stability of learning by studying the change in *DI* as more inputs were presented after 1 h of training. To test this, the duration of presentation of the inputs was doubled from 1 to 2 h. During this time, the inputs were once again sampled at random from the training set and presented for a duration that was selected from an exponential distribution of 30 ms.

**Figure 9:**
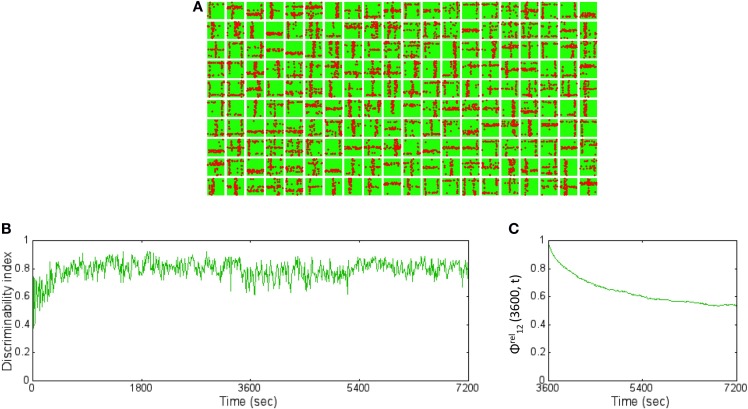
**The basis vectors and basis set learning after 2 h of training on initial training data set. (A)** The synapses between the source neurons and each *E* neuron in the reservoir form receptive fields (similar to the ones shown in Figure 4B) but slightly modified after 2 h of training compared to after 1 h of training. **(B)** The *DI* is steady throughout the extra 1 h of training hovering around 0.8. This implies that the learning has stabilized causing the discriminability to be stable as well. **(C)** The relative synaptic distance between *E→ E* synapses from source layer neurons to the reservoir neurons was compared to randomly shuffled synapses or synapses formed due to chance. The slope of the distance trace slowly decreases suggesting stability in learning while the final value of 0.6 suggests that the learning stabilizes the network to a state that is far from chance (see text for further details).

The readout codes for all patterns in the training set after 1 h and after 2 h were compared. The ternary codes for each pattern were compared and the codes do no match at all. This change is partially explained by subtle changes in the receptive fields (Figure 9A) compared to after 1 h (Figure 4A). The *DI* is very stable and hovers around 0.8 (Figure 9B) throughout the extra hour of training. To measure the change in synapses more precisely, the relative synaptic distance Equation (19) between layer 1 and layer 2 synapses after 1 and 2 h of training. This relative distance ϕ*^rel^*_12_(3600, *t*) was tracked once every 10 s from 1 to 2 h (Figure 9C). The plot shows that the distance slowly changes during the first hour and stabilizes to ~0.6. This implies that the synaptic weight changes in a more meaningful fashion compared to changes due to pure chance. Furthermore, the rate of change of the relative synaptic distance during the hour is slow and thus implies a stable regime of synaptic adaptation.

The selectivity of the readout neurons to a subset of the reservoir neurons emerges from E-STDP based on pattern of firing in the reservoir. Once the selectivity is established for the readout neurons, it does not change very much during the second hour of training. This is evident from the observation that the synaptic distance between the two layers does not change (Figure 5C) and that all the synapses to readout neurons become fully potentiated with a unimodal distribution of synaptic strengths. The synaptic strengths between the reservoir neurons and the readout neurons are unimodal by the end of 1 h of training due to E-STDP and continue to remain stable during the extra hour of training. This is reflected in ϕ*^rel^*_23_(3600, *t*) that is not defined since the ϕ_23_(3600, *t*) = ϕ*^shuffled^*_23_(3600, *t*) for all *t* after 3600 s. This is because the synapses between the *E* neurons in the reservoir and the *E* neurons in the sink layer do not change (Figure 5C) for reasons explained earlier. This means that ϕ*^rel^*_23_(3600, *t*) is not defined for all *t*.

**Figure 10:**
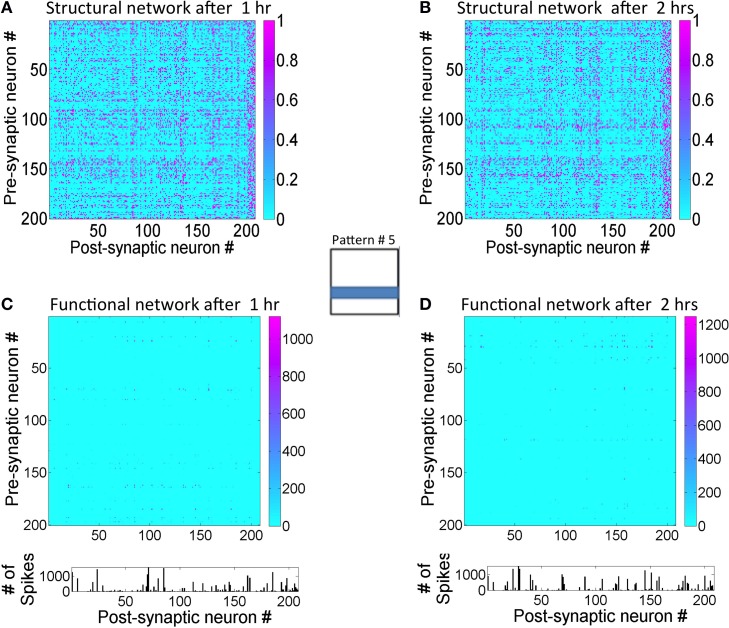
**State-dependent computing in the network can be observed by tracking activity within the reservoir layer. (A)** The graph showing the strength of the *E→E* synaptic connections between the reservoir neurons that are connected to readout units #1 and #8 after 1 h of training. These readout units are maximally active during the presentation of pattern #5 for *d* = 1.4 s at the source neurons. The normalized synaptic (obtained by dividing *g* by *g_max_*) strengths are between 0 and 1. This set of network connections between reservoir neurons is referred to as a structural network. **(B)** The structural network for the same input pattern after 2 h of training is shown here. Synaptic plasticity does alter the structural network as the learning progresses. **(C)** The state transitions between the neurons in the graph are tracked and plotted after 1 h of training to show the functional network in action during the processing of input pattern #5. The functional network is sparse compared to the structural network. The relative strengths of transitions between reservoir neurons during the presentation of pattern #5 for *d* = 1.4 s period can be assessed using the firing rates of the reservoir neurons. The neurons #6, #28, #63, #75, #155, #157, and #177 all have higher relative firing rates than other reservoir neurons in the graph. **(D)** The functional network after 2 h of training shows a functional network has changed compared to the one after 1 h. Neurons #25, #31, #145, and #150 are now the most active.

The lateral connectivity in the reservoir causes a state dependent firing regime (Buonamano and Maass, 2009). To understand this better for the proposed network, used a *back-trace* approach was adopted as follows. The readout neurons that fired with a maximum firing rate for a given input pattern were first selected. The neurons back-trace to the reservoir from these readout neurons were then identified. For example, the readout neurons #1 and #8 fired with a ternary code of 2 were first selected. The synaptic connections between the reservoir neurons are represented in the graph (Figure 10A after 1 h and Figure 10B after 2 h). It should be noted that the strong connections between the reservoir neurons only depict the anatomical or structural aspect of the network. The resulting set of reservoir neurons and their connections between each other and the two readout units is referred to as a *structural* network. The strong connection between the *E* reservoir neurons are not necessarily unique to the selected readout neurons since other readout neurons that are active for other inputs may also be connected to some of the same reservoir neurons found in the structural network. Similarly, the readout neurons are not unique to the input pattern since the code in the sink layer is distributed. This means that the same readout neuron could fire as part of another readout code that represents a different input pattern.

When the structural network is tracked temporally for the duration of input presentation (i.e., for *d* = 1.4 s), the network dynamics shows that a select subset of reservoir neurons fire in a complex spatiotemporal sequence. A state transition graph can be plotted from this firing sequence (Figure 10C). This graph is referred to as the *functional* network that is very sparse when compared to the structural network. After further training for an additional hour, the functional network for the same pattern changes (Figure 10D). Here the transition frequencies between some of the neurons are somewhat reduced compared to the functional network after 1 h. This implies that the network is able to sharpen the neural trajectory further with training.

The state-transitions at the reservoir combined with stable connections between the reservoir and output neurons means that the ternary code at the readout neurons will change based on these transitions in the reservoir. These changes in the neural trajectory cause the ternary code in the readout units to be different even for identical inputs. This implies that repeatable readout neuron activity (in response to input patterns) is not achievable in this network. However, the *relative* codes between a pattern and the rest as computed by the *DI* are stable (Figure 9B).

### Plasticity to new inputs after initial learning

**Figure 11:**
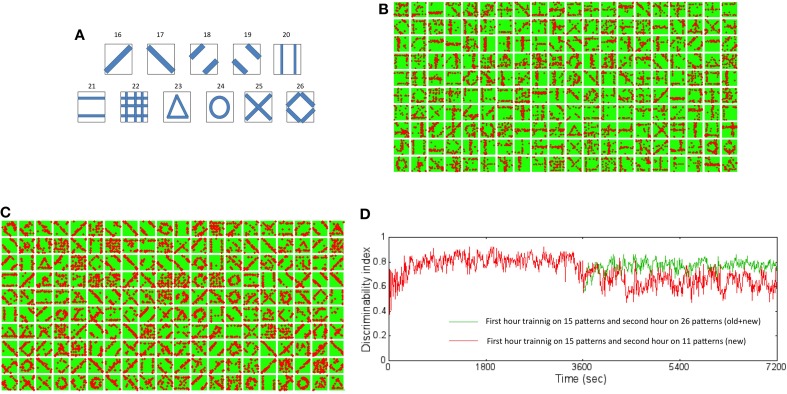
**New training set data set and resulting receptive fields formed due to learning for two different training regimes. (A)** The new training data set is composed of 11 new patterns not in the original data set. These patterns were added to the original data set and then used for training the network to test the ability of the network to learn new information without forgetting old information. **(B)** The new receptive fields formed by training for an additional hour with both old and new data after initial training on the original data set for an hour is shown here. The new receptive fields show newly learned features that incorporate features such as a diagonal line (for example, neuron #4 and #18). **(C)** The new receptive fields formed by training for an additional hour with only new data after initial training on the original data set for an hour is shown here. The new receptive fields change dramatically from the original set (see Figure 4B) with mostly features that reflect the new patterns and very little from the old patterns. **(D)** The *DI* was compared for the two training regimes. The *DI* was retained at a high value of 0.8 (green trace) when the network was exposed to both old and new patterns in the second hour of training. The network however was found to have a lower *DI* value of 0.62 (red trace) on average suggesting forgetting of old information. However, the interesting aspect is that *DI* decreases gradually suggesting that the network does not loose the ability to discriminate between old patterns or between old and new patterns abruptly or “catastrophically” but in a more graceful manner.

In order to study the capacity of the network to learn new inputs, a second training set with new input patterns was added to the initial training data set (Figure 11A). The network that was trained with the initial training data for 1 h was presented with the both old and new inputs for an additional hour. The receptive fields show slow adaptation to the new features while retaining features from the old patterns as well (Figure 11B). For example, the learned receptive for reservoir neuron #4 (fourth box from the left on top row in Figure 11B) at the end of 2 h shows a diagonal set of synaptic connections that was absent after training the network with the initial training set (Figure 4B). On the other hand, the neuron #18 (third box from the right on the top row in Figure 11B) remains very similar to the receptive field learned after 1 h (Figure 4B).

The network was also studied for their ability to learn new patterns when they were presented with only new patterns during the second hour of training. This test was more stringent than the first experiment above and provides a more precise picture of the network's ability to retain past information while learning new information. The changes in the receptive fields reflect rapid re-learning with adaptations to features found in the new inputs (Figure 11C) after 2 h of training compared to receptive fields after training for 1 h with the initial training set (Figure 4B). For example many of the receptive fields show diagonal and circular features. It is noteworthy that very few receptive fields now reflect the initial training set.

The *DI* was computed for the above two cases of training. When the network is presented with both old and new patterns during the second hour of training, the *DI* was very stable relative at ~0.8 when tested on all the 26 patterns after 2 h of training relative to the first hour of training (the green trace in Figure 11D). This implies that the network is able to discriminate both the old and new patterns after 2 h of training. In comparison, when the network was trained only with new patterns, the *DI* falls to a lower value of ~0.6 (the red trace in Figure 11D). This implies that the network is not as discriminatory as in the first case implying that the network forgets. However, the network does not exhibit catastrophic forgetting (French, 1994). Catastrophic forgetting occurs when the network abruptly (i.e., in a few time steps) and completely (i.e., with very poor discrimination) forgets previously learned patterns in exchange for learning new patterns. Since the DI only degrades from ~0.8 after 1 h to ~0.6 after an additional hour of training only with new patterns, the network does not abruptly forget the old patterns. This graceful and slow degradation in DI shows that the network gradually forgets previously learned information but not catastrophically.

**Figure 12:**
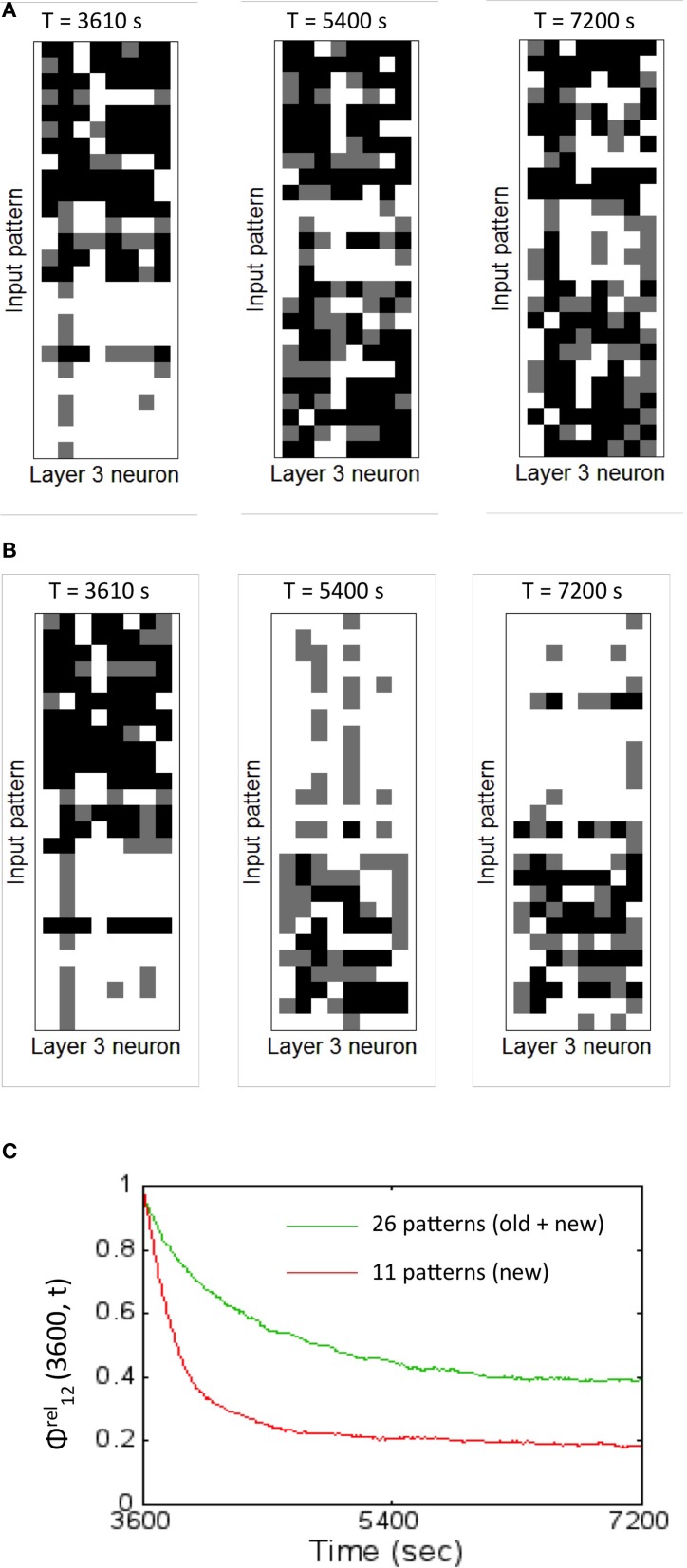
**The readout codes generated by the network for two different training regimes shows the network is plastic to new inputs while also being stable to old information. (A)** The readout codes generated for the network when trained on all the 26 patterns during the second hour of training. The testing was performed with all the 26 patterns. The readout code is washed out in the beginning for the new patterns but slowly is assimilated by the network generating a rich readout code for all the 26 patterns after 2 h. The resulting *DI* is stable (as shown in Figure 11) suggesting robust discrimination. **(B)** The readout codes generated for the network when trained on only on the 11 new patterns during the second hour of training. The testing was performed with all the 26 patterns. The readout code is washed out in the beginning for the new patterns but the network rapidly learns the new features suggesting the highly plastic nature of the network. The readout code starts to wash out for the 15 old patterns after 2 h. The resulting *DI* is still strong enough (as shown in Figure 11) suggesting slow forgetting of old information. **(C)** The relative synaptic distance between *E* → *E* synapses from source layer neurons to the reservoir neurons was compared to randomly shuffled synapses or synapses formed due to chance for the two training regimes are shown. The slope of the relative distance trace slowly decreases (green trace) for the case where the old and new patterns are presented suggesting stability in learning. However, the slope changes more dramatically (red trace) for the case when the network is trained only on the new patterns. The final value of 0.5 (old + new) and 0.2 (for new only) suggests that the learning stabilizes the network to a state that is different from pure chance.

The readout codes for the two cases provide some more insight into the network performance. The network begins at the same starting point (i.e., after training with 15 patterns for 1 h). The readout codes are very different for the two cases after 2 h. When the network is trained with both old and new patterns, the readout code for the 26 patterns appears with much more variations (Figure 12A) compared to the case when trained only with new patterns. In the latter case, the network appears to have more washed out codes for the old patterns compared to the new patterns (Figure 12B). This confirms that the network forgets the old patterns in the second case compared to the first case. This is to be expected to some extent because the network is plastic and is expected to learn the new inputs as it experiences that more than the old patterns. However, it is noteworthy that the network does not exhibit catastrophic forgetting as discussed above. This shows that the network is able to assimilate the old information along with the new information to create a new readout codes such that the resulting *DI* is sufficient for discrimination between all patterns (old and new) at least for some time (in this case for about an hour). Understanding how this could be extended is a subject for future study.

The relative synaptic distance ϕ*^rel^*_12_(3600, *t*) was tracked once every 10 s from 1 to 2 h between the receptive fields for the two cases of training. The distance changes slowly for the case when the network is presented with old and new patterns (the green trace in Figure 12C). In comparison, the weight changes are far more drastic (the red trace in Figure 12C). Here the rate of change is steep implying that the network undergoes sharp changes during the early learning phase in the second hour but then stabilizes to a non-zero value. This implies that the network undergoes synaptic changes due to learning driven by STDP based on new training data as opposed to changes due to pure chance. The synapses between the *E* neurons in the reservoir and the *E* neurons in the sink layer do not change (similar to Figure 5C) for reasons explained earlier. This means that ϕ*^rel^*_23_(3600, *t*) is not defined for all *t*.

**Figure 13:**
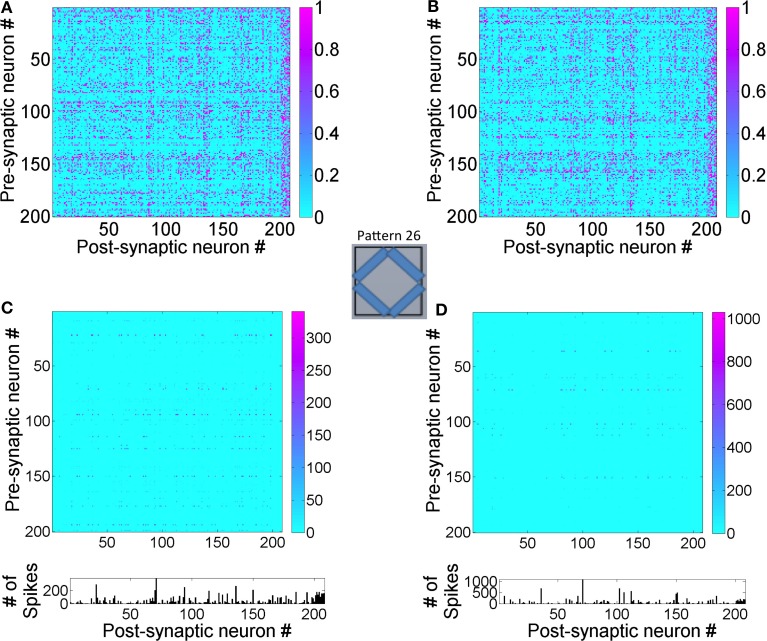
**State-dependent computing in the reservoir layer is affected by new training data. (A)** The structural network connected to readout units #4 and #8 after 1 h of training. These readout units that are maximally active during the presentation of the new pattern #26 for *d* = 1.4 s. **(B)** The structural network for the combined training data of old and new input patterns after 2 h of training is shown here. Synaptic plasticity alters the structural network as expected. **(C)** The transitions between *E* → *E* neurons in the reservoir after 1 h of training This functional network shows firing activity at many more neurons and this is manifested by the novelty of the new pattern that the network has never processed before. **(D)** This functional network after 2 h of training is sparser compared to the one shown after 1 h since the network is able to learn the new pattern by adapting its receptive fields and generating new readout codes that are more discriminatory.

In order to understand how this occurs, the lateral connectivity of the graph was analyzed. A pattern from the new data set was selected for analysis (Figure 13) and presented to the source neurons for *d* = 1.4 s. Since the first hour of training is based on the initial training set, the network was never exposed to this new pattern before. The readout neurons (#4 and #8) that fired maximally for the new pattern was identified and its structural network was identified at the 1 h mark (Figure 13A).

The structural network selected by these two neurons changes between 1 and 2 h when the network is trained with both old and new patterns (Figure 13B). When the functional network was extracted after the first hour of training, the network dynamics shows that many reservoir neurons are accessed in a complex spatiotemporal sequence (Figure 13C). This is because the network attempts to process the unknown input pattern using as many receptive fields as possible. Once the network is trained for an hour more with both old and new patterns, the network is able to readout using a relatively sparse neural trajectory (compare Figures 13C,D). This is because the basis set adapts to incorporate new features in the new input pattern data set during the second hour of training. This modifies the functional network due to changes in firing rates of some neurons in the graph (see in Figures 12C,D) as well as changes in the functional network. The functional network for the case when trained only on new patterns in the second hour is similar to Figure 12D except that the spatiotemporal trajectories in the network are biased toward the new inputs as opposed to the network that is exposed to both the old and new patterns.

## Discussion

The interaction between E-STDP and I-STDP enables spiking neuronal networks to learn to discriminate patterns in a self-organized fashion. A hallmark of self-organizing systems is a composition of relatively “dumb” units connected together and constrained by “interaction dominant dynamics” (Ihlen and Vereijken, 2010). In the case of the simulated network presented here, the connection strength between neurons is always altered by synaptic plasticity, effectively changing the network topology. The structure of the network is tuned so as to enable uncorrelated neurons that are randomly connected to become correlated in a balanced way so as to produce meaningful network-level behavior.

Balance in the network is at the level of excitatory and inhibitory currents. These currents are observed to balance each other, leaving the resultant current near zero. In the present model, changing synaptic conductance via STDP for inhibitory and excitatory synapses helps achieve this balance. There are other models (Vogels et al., 2011; Srinivasa and Jiang, 2013) that also have explored the effects of interaction between these two types of STDP for memory formation and stability but have not explored the question of unsupervised discrimination of patterns.

In a recent model, the synaptic efficacy via shot-term plasticity (Klampfl and Maass, 2013) was used to achieve stable and balanced networks but that work also did not look into unsupervised pattern learning. In other biologically plausible networks, synaptic connections can be created or destroyed also known as structural plasticity (Leuner and Gould, 2010). But without that option, plasticity is left as the only possible mechanism for change within the network.

Networks without I-STDP fail to reach a balanced state for any of a large set of possible parameters. It is not only a practical matter that inhibitory STDP is required, but there are deep connections to self-organizing systems as well. Self-organization is usually the result of two opposing effects. In the strong cases, these opposing effects are some mutually-referring function of each other (Nicolis and Prigogine, 1977; Witten and Sander, 1981). Here, excitatory and inhibitory STDP play these roles, and together produce various forms of compensatory feedback (Luz and Shamir, 2012). It should be noted that the obtained results are based on one of many possible I-STDP functions found in the brain (Vogels et al., 2013) and not all of them will necessarily result in a current balance. A recent article explored the distinct I-STDP window shapes in tuning neuronal responses (Kleberg et al., 2014). The exact role of each shape of I-STDP function on brain function remains to be explored in the future.

This memory trace or neural trajectories in the reservoir evolve both in space and time (Rabinovich et al., 2008; Buonamano and Maass, 2009; Buzsáki, 2010). It is known that discriminating between several trajectories requires complex mechanisms with many dedicated readers (Jortner et al., 2007; Masquelier et al., 2009; Buzsáki, 2010). Our approach proposes an algorithm for computing *DI* that can help discriminate between input patterns but this is still not a biologically plausible mechanism. Using the *DI* metric to probe network dynamics shows that the proposed network can discriminate between patterns that form complex neural trajectories. Furthermore, this discrimination is not susceptible to catastrophic forgetting.

It should be noted the order of presentation of the input patterns to the network at the source neurons provides different contexts and changes the state-dependent firing patterns in the network. This causes the ternary code in the readout units for identical inputs to be different. However, the *relative* firing rate code as computed by the DI metric is invariant to the order of input pattern presentation (not shown).

The *DI* measure derived in this work is related to information theory. Information theory informs us about the amount of information a neural response (by sink layer neurons) carries about the stimulus (source layer neurons). In this theory, information is quantified using entropy measures. The *DI* measure captures the amount of information about the stimulus in the neural response and is thus closely associated to mutual information (Borst and Theunissen, 1999). In the *DI* measure, separability is closely linked to entropy measures. For example, *D_inter_* is associated to noise entropy since it provides a measure of variability in neural response to the same input stimulus while *D_intra_* is associated to response entropy as it measures variability in neural response to all the stimulus types presented to the network. Uniqueness on the other hand is directly linked to response entropy as it measures the variations in all possible neural responses to all possible stimulus conditions. This could be a useful future direction for further investigation.

The network design proposed in this paper for unsupervised discrimination has two key features that enable fault-tolerant properties in a manner similar to our previous work on self-supervised learning of spatiomotor transformations (Srinivasa and Cho, 2012). The first feature is the reduction of number of spiking neurons from layer 1 to layer 2 (i.e., *K* > *N*). This allows the network to compress the input features into an encoding consisting of a smaller subset of neurons in layer 2. The absence of spiking activity from some input neurons can still be tolerated due to inputs from neighboring input neurons within the reservoir. The second is the recurrent STDP connections between neurons within layer 2. With this feature, the spiking activity due to neighboring neurons within layer 2 enables STDP to eventually strengthen synapses between the neurons that receive inputs from layer 1 and weaken those that do not receive any inputs from neurons in layer 1. This feature might provide robustness against the complete loss of spiking activity within neurons because recurrent connections within the reservoir might enable neurons that do not receive any feed-forward input from layer 1 to still propagate spiking activity to layer 3. Thus, the network could exhibit tolerance to complete loss of spiking activity in the input neurons.

**Figure 14:**
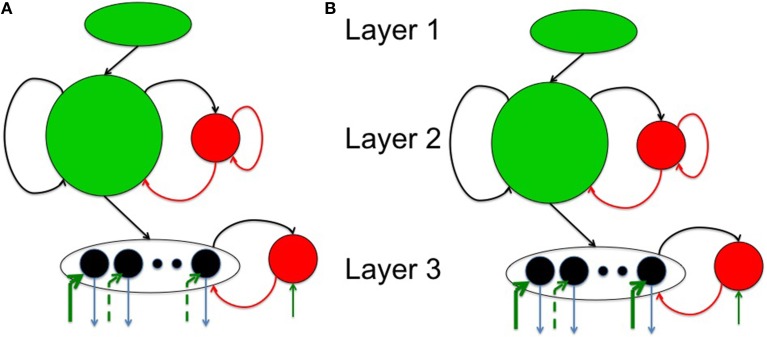
**Two plausible neural mechanisms to enable supervised learning by extending the network architecture presented here. (A)** The first possibility is to replace the sink layer architecture of the original network with a new one as shown here. Here each label (thick green arrow) corresponds to each pattern that is to be classified. Black lines represent excitatory synapses while red ones correspond to inhibitory inputs. Green arrows represent excitatory inputs from external sources. **(B)** The second possibility assumes a readout code that is more distributed (more than one thick green arrow—see text for more details).

It may be possible to extend the proposed architecture for unsupervised discrimination to learn using supervisory labels. Two possible mechanisms are briefly explored here. In the first case, each readout neuron in layer 3 can be stimulated by an externally provided spike train that corresponds to a label for the input pattern presented in layer 1 (Figure 14A). Here each readout neuron uniquely codes for one label thus requiring as many readout neurons as labels. This external input can be considered as the top-down (TD) input while the spikes from the reservoir neurons to the readout neurons can be considered as bottom-up (BU) inputs.

When an external label is available, it can cause TD stimulation of the appropriate readout neuron even if there are no BU inputs since it is assumed that these TD inputs are provided via very strong synapses (reflected by thick green arrows in Figure 14A). The TD inputs increase the inhibition to the readout neurons thus creating network dynamics in the readout akin to a winner take-all network. It should be noted that the inhibitory neurons in layer 3 is constantly stimulated by weak external spiking inputs (think arrow to the red circle in Figure 14A). When the readout neurons are stimulated by both BU and TD inputs, the synapses from the reservoir neurons to the readout neurons could be strengthened due to E-STDP. At a later time, if the label is removed, the readout neurons can generate peak activity in the readout neuron corresponding to the correct label. But as observed before, the readout codes change slowly and constantly due to plasticity in the reservoir. This can result in misclassification errors. These errors can however be fixed by periodically providing the correct labels that can trigger STDP based learning to quickly correct the mistakes by retuning the synapses from the reservoir to the readout neurons.

In the second case, the readout neurons could represent a distributed code (Figure 14B). In this case, the joint spiking activity of the entire population of readout neurons represents a label for each input. This network can also be trained via labels at the appropriate readout neurons as described above and the learning process due to BU and TD stimulation of the readout neurons can cause the readout neurons to generate correct answers even when the teaching labels are removed. This network is however also susceptible to constant forgetting thus requiring periodic stimulation by the external supervisory sources to correct the mistakes made by the readout neurons in classifying input patterns.

The functional network response of the network to inputs via neural trajectories in the reservoir indicates the use of a distributed code with many reservoir neurons being activated during the input presentation. Normally the relative firing rates between reservoir neurons (i.e., the number of times a neurons fires relative to other neurons in the reservoir) is not high. However, in some cases a single neuron in the reservoir may exhibit a high relative firing rate. Thus, some neurons in the reservoir can encode for the entire input in some cases while at the same time require neural trajectories to encode other inputs. This flexibility is a hallmark of neural systems where single neurons (also known as *grandmother cells*) are known to encode for objects (Perrett et al., 1982; Rolls, 1984; Yamane et al., 1988; Quiroga et al., 2005) while there are other concepts that require distributed codes with complex neural trajectories (Rabinovich et al., 2008; Buzsáki, 2010).

The most interesting aspect of our network is that it is able to discriminate between old patterns already presented in an initial training session while also adapting to new patterns without losing its ability to discriminate among old patterns. The learned connectivity especially between the source layer neurons and the *E* neurons in the reservoir appears to be critical for this capability as exemplified from the control experiments. The ternary code appears to produce the highest *DI* metric compared to other codes since the inter-pattern distance is the lowest for the ternary code compared to the case with other codes. The network does not exhibit catastrophic forgetting and is more robust if exposed to old patterns occasionally during the learning of new patterns. Incorporating the means to achieve a homeostatic balance due to an interaction between inhibitory and excitatory STDP in all these networks may well enable self-organized discrimination of patterns while exhibiting the requisite dynamics to address the stability vs. plasticity dilemma.

### Conflict of interest statement

The authors declare that the research was conducted in the absence of any commercial or financial relationships that could be construed as a potential conflict of interest.

## Figures and Tables

**Figure A1:**
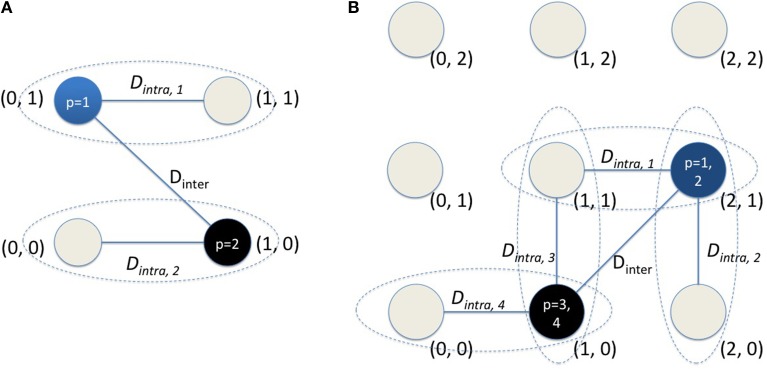
**The details of *DI* computation for two different examples are shown here. (A)** The readout code for the first example uses *M* = 2 and *C* = 2 as visualized here. Each node represents one of four possible states. There are a total of input patterns (*P* = 2) for this example. **(B)** The readout code for the second example uses *M* = 2 but *C* = 3 as visualized here. Each node represents one of 9 possible states. There are a total of 4 input patterns (*P* = 4) for this example.

## Appendix

### Examples for DI computation

Let us assume that the coding level *C* = 3 for both the examples below and further let us assume that there are two readout units in the sink layer of the network. This allows us to visualize the readout codes in 2-D for simplicity. This analysis however readily extends to other coding levels.

**Example 1:** Assume that there two test patterns (*P* = 2) and each of them is presented 10 times resulting in 10 readout codes for each test pattern as follows: S^1^ = {(0, 1); (0, 1); (0, 1); (0, 1); (1, 0); (1, 1); (1, 1); (0, 1); (0, 1); (0, 1); (0, 1)} and S^2^ = {(1, 0); (1, 0); (0, 0); (0, 0); (1, 0); (1, 0); (0, 0); (1, 0); (0, 0); (1, 0)}. Since there are two patterns and *C* = 2, there are four possible readout codes in general (Figure A1A). But based on S^1^ and S^2^, the two readout codes cluster around (0, 1) for *p* = 1 and (1, 0) for *p* = 2.

The various values for *DI* can be computed as follows. Using Equation (10) and the readout code S^1^, *D*_*ientra*, 1_ = 6^*^4/(10^*^9/2) = 24*M*/45 and *D*_*intra*,2_ = 8^*^2/(10^*^9/2) = 16*M*/45; thus the average *D_intra_* = 20*M*/45. Since the readout codes cluster around (0, 1) and (1, 0) for the two test patterns, *D_inter_* = 2*M*. Using these *D_inter_* and *D_intra_*, separability can be computed as ε = 1−10/45 = 35/45. Since there are two unique codes for the two test patterns, uniqueness γ = 1. Thus, the discriminability index for this example will be *DI* = 35/45 = 0.78.

**Example 2**: Assume that there are four test patterns (*P* = 4) and each of them is presented 10 times resulting in 10 readout codes for each test pattern as follows: S^1^ = {(2, 1); (2, 1); (2, 1); (2, 1); (2, 1); (1, 1); (1, 1); (2, 1); (2, 1); (2, 1); (2, 1)}, S^2^ = {(2, 1); (2, 1); (2, 0); (2, 0); (2, 1); (2, 0); (2, 1); (2, 1); (2, 1); (2, 1)}, S^3^ = {(1, 0); (1, 0); (0, 0); (0, 0); (1, 0); (1, 0); (0, 0); (1, 0); (0, 0); (1, 0)}, S^4^ = {(1, 0); (1, 0); (1, 1); (1, 0); (1, 0); (1, 1); (1, 0); (1, 0); (1, 1); (1, 0)}. This scenario can be visualized using four possible readout codes in general (Figure A1B). Based on four readout codes, they cluster around (2, 1) for *p* = 1, 2 and (1, 0) for *p* = 3, 4.

The various values for *DI* can be computed as follows. Using Equation (10) and the readout code S^1^, *D*_*intra*, 1_ = 16*M*/45, *D*_*intra*, 2_ = 21*M*/45, *D*_*intra*, 3_ = 24*M*/45, *D*_*intra*, 4_ = 21*M*/45. Thus, the average *D_intra_* can be computed as 41*M*/90. Since the readout codes cluster around (0, 1) and (1, 0) for the four test patterns, the *D*_*inter*_ = 2^*^2^*^*M*/(4^*^3/2) = 2*M*/3. Using these *D_inter_* and *D_intra_*, separability can be calculated as ε = 1−41/60 = 19/60. Since there are only two unique codes for the four test patterns, uniqueness γ = 1/2. Thus, *DI* for this example will be *DI* = (19/60)^*^(1/2) = 0.16. This is lower compared to Example 1 since the four patterns are less separable compared to the two test pattern case and the readout codes are also not unique enough compared to Example 1.

These examples illustrate the basics of how the *DI* is computed and can be readily extended to deal with networks that have larger *M* and are coded with different coding levels.
